# Temperature‐Related Responses of an Invasive Mussel and 2 Unionid Mussels to Elevated Carbon Dioxide

**DOI:** 10.1002/etc.4743

**Published:** 2020-06-27

**Authors:** Diane L. Waller, Michelle R. Bartsch, Eric G. Lord, Richard A. Erickson

**Affiliations:** ^1^ US Geological Survey Upper Midwest Environmental Sciences Center La Crosse Wisconsin

**Keywords:** Toxic effects, Invasive species, Mollusk toxicity, Dreissenid, Unionid, Acidification

## Abstract

Zebra mussels (*Dreissena polymorpha*) have exacerbated the decline of native freshwater mussels (order Unionida) in North America since their arrival in the 1980s. Options for controlling invasive mussels, particularly in unionid mussel habitats, are limited. Previously, carbon dioxide (CO_2_) showed selective toxicity for zebra mussels, relative to unionids, when applied in cool water (12 °C). We first determined 96‐h lethal concentrations of CO_2_ at 5 and 20 °C to zebra mussels and responses of juvenile plain pocketbook (*Lampsilis cardium*). Next, we compared the time to lethality for zebra mussels at 5, 12, and 20 °C during exposure to partial pressure of CO_2_ (PCO_2_) values of 110 to 120 atm (1 atm = 101.325 kPa) and responses of juvenile plain pocketbook and fragile papershell (*Leptodea fragilis*). We found efficacious CO_2_ treatment regimens at each temperature that were minimally lethal to unionids. At 5 °C, plain pocketbook survived 96‐h exposure to the highest PCO_2_ treatment (139 atm). At 20 °C, the 96‐h lethal concentration to 10% of animals (LC10) for plain pocketbook (173 atm PCO_2_, 95% CI 147–198 atm) was higher than the LC99 for zebra mussels (118 atm PCO_2_, 95% CI 109–127 atm). Lethal time to 99% mortality (LT99) of zebra mussels in 110 to 120 atm PCO_2_ ranged from 100 h at 20 °C to 300 h at 5 °C. Mean survival of both plain pocketbook and fragile papershell juveniles exceeded 85% in LT99 CO_2_ treatments at all temperatures. Short‐term infusion of 100 to 200 atm PCO_2_ at a range of water temperatures could reduce biofouling by zebra mussels with limited adverse effects on unionid mussels. *Environ Toxicol Chem* 2020;39:1546–1557. Published 2020. This article is a U.S. Government work and is in the public domain in the USA. *Environmental Toxicology and Chemistry* published by Wiley Periodicals LLC on behalf of SETAC.

## INTRODUCTION

Dreissenid mussels have posed an aquatic invasive species challenge in the United States since their arrival in the Great Lakes in the 1980s (Griffiths et al. 1991). Zebra (*Dreissena polymorpha*) and quagga (*Dreissena bugensis*) mussels are voracious filter‐feeders with high reproductive capacity, resulting in altered nutrient cycles, shifts in trophic structures, and extirpation of some native species in systems where they have established (Vanderploeg et al. 2002; Higgins and Vander Zanden 2010; Bootsma and Liao 2014; Mayer et al. 2014). Native unionid mussel (order Unionida) populations have been particularly decimated by the more competitive and prolific zebra mussel (Nalepa et al. 1996; Ricciardi et al. 1998; Martel et al. 2001; Strayer and Malcom 2018). Efforts to manage dreissenid populations have focused primarily on chemical tools, especially oxidizing chemicals (i.e., bleach; Glomski 2015), potash (Department of Fisheries and Oceans 2014; Fernald and Watson 2014), copper‐based compounds (Offutt Air Force Base 2009; Claudi et al. 2014; Hammond and Ferris 2019), and the biopesticide Zequanox® (Molloy et al. 2013b; LimnoTech 2019). Though effective, these options can be expensive to apply, toxic to native species, persistent in the environment, and a source of harmful by‐products (Mackie and Claudi 2010). Control options for dreissenids that are safe for native mussels are especially limited. Zequanox is relatively safe to nontarget species (Molloy et al. 2013a; Meehan et al. 2014; Luoma et al. 2015; Weber et al. 2015; Waller et al. 2016; Waller and Luoma 2017), but it has limited efficacy at water temperatures <15 °C (Luoma et al. 2018).

Carbon dioxide has received increased attention for use in aquatic invasive species management to deter fish (Kates et al. 2012; Donaldson et al. 2016; Cupp et al. 2017a) and as a biocide for American bullfrogs (*Lithobates catesbeianus*; Abbey‐Lambertz et al. 2014), New Zealand mud snails (*Potamopyrgus antipodarum*; Nielson et al. 2012), invasive red swamp (*Procambarus clarkii*), rusty crayfish (*Faxonius rusticus*; Fredricks et al. 2020), and nuisance fish (Cupp et al. 2017b). Carbon dioxide has several advantages over traditional chemical control tools for aquatic invasive species; it costs less, is widely available, is easy to apply, lacks harmful chemical residues, and has low risk to human health (Treanor et al. 2017). Previously, CO_2_ was found to reduce attachment and induce mortality of zebra mussels when 100% CO_2_ gas was bubbled into water (McMahon et al. 1995; Payne et al. 1998). Waller and Bartsch (2018) found that exposure to 400 mg L^−1^ CO_2_ for 72 to 96 h at approximately 12 °C was efficacious for adult zebra mussels. The same study found that effective treatment regimens for zebra mussels did not cause significant mortality of a native mussel (fatmucket, *Lampsilis siliquoidea*), indicating that CO_2_ could be used to selectively control dreissenids in a unionid habitat.

Carbon Dioxide‐Carp is registered as a pesticide by the US Environmental Protection Agency (USEPA) for deterrence of Asian carp and to control aquatic nuisance species when applied under ice (US Environmental Protection Agency 2019). The current registration could also allow for the use of CO_2_ to kill zebra mussels in water bodies during periods of ice cover, with appropriate US state approvals and discharge permits; however, first efficacious treatment regimes in cold water need to be determined.

When CO_2_ dissolves in water, it reacts to form carbonic acid, which dissociates into free CO_2_, bicarbonate, and carbonate, the relative proportions of which are dependent on alkalinity and temperature (Robbins et al. 2010). When water temperature changes, the partial pressure of carbon dioxide (PCO_2_) is calculable from water chemistry parameters; but the temperature‐related response of an organism to CO_2_ is less predictable. Carbon dioxide diffuses into the plasma or hemolymph, and physiological and behavioral responses will be dependent on the animal's buffering capacity as well as respiratory and metabolic compensation (Treanor et al. 2017). Unionids and dreissenids occupy the same macrohabitat and compete for food, but as more recent invaders of freshwater, the latter are less tolerant of environmental extremes (e.g., hypoxia, osmotic and pH shifts, and hypercapnia; McMahon 1991, 1996; Dietz et al. 1996; Byrne and Dietz 2006; Garton et al. 2014; Waller and Bartsch 2018). Dreissenids are epifaunal, are fully exposed to the water column, and attach to substrates by byssal threads. In contrast, unionids are infaunal, and burial position can vary with season (i.e., temperature change), reproductive activity, disturbance (Amyot and Downing 1997, 1998; Waller et al. 1999; Watters et al. 2001; Block et al. 2013), and in response to stressors (Gagnon et al. 2004; Archambault et al. 2014; Waller and Bartsch 2018; Waller et al. 2018). These behavioral and physiological differences could be exploited to selectively control zebra mussels, especially in unionid habitats.

We compared responses of zebra mussels and 2 unionid mussels to CO_2_ across a temperature range to determine treatment scenarios that had the greatest efficacy to invasive mussels and safety margin to unionid mussels. Two independent trials were conducted. The first established lethal concentrations (LCs) of CO_2_ in 24‐ to 96‐h exposures to produce 50, 75, and 99% mortality (LC50, LC75, and LC99 values) of zebra mussels at 5 and 20 °C and compared survival and behavioral response of juvenile plain pocketbook (*Lampsilis cardium*). The second trial estimated the lethal time exposure duration to produce 99% mortality (LT99 values) of zebra mussels at a fixed PCO_2_, as determined in trial 1, at 5, 12, and 20 °C. In addition, the lethal and sublethal effects of maximum CO_2_ exposure were measured in juvenile plain pocketbook and fragile papershell (*Leptodea fragilis*).

## MATERIALS AND METHODS

### 24‐ to 96‐h lethal concentration trials at 5 and 20 °C

#### LC—test system

Trials were conducted in a proportional flow‐through diluter system at 5 and 20 °C to determine effective CO_2_ concentrations for preventing attachment and inducing mortality of zebra mussels (Waller and Bartsch 2018). The diluter included a mixing box that delivered water to a serial dilution box, partitioned into 6 chambers to deliver 5 exposure concentrations plus a control (Supplemental Data, Figure S1). Air stones were submerged into the first chamber and delivered CO_2_ gas from compressed gas cylinders at a predetermined flow rate to produce the highest targeted CO_2_ concentration in the 20 °C trial. Flow rate was held constant in the 5 °C trial to compare the resultant PCO_2_ levels at the 2 test temperatures. Carbon dioxide concentration was diluted by approximately 20% with untreated water in each subsequent diluter chamber and categorized as high, medium‐high, medium, medium‐low, and low. Each diluter chamber delivered CO_2_‐treated water to 4 replicate test tanks and clean, untreated water from the mixing box outflowed to 4 control tanks. Test tanks (20‐L glass aquaria) were filled to a volume of 13 L and received a continuous supply of temperature‐controlled well water at a rate of 150 mL/min throughout the test period. We chose exposure durations of 24 to 96 h, which were similar to those tested at 12 °C by Waller and Bartsch (2018). Carbon dioxide was infused into the test system for 96 h. Partial pressure of CO_2_ (1 atm = 101.325 kPa) in each tank was calculated from daily measurements of pH, temperature, and alkalinity using the US Geological Survey's CO2calc program (Robbins et al. 2010).

#### LC—Test animals

Postsettlement (juvenile) and adult zebra mussels were hand‐collected from Lake Minnetonka, Minnesota, USA, in November 2016 for the 5 °C trial and from White Bear Lake, Ramsey County, Minnesota, USA, in October 2017 for the 20 °C trial. Zebra mussels were transferred to 50‐L flow‐through tanks with a well water supply and acclimated to the test temperature (either 5 or 20 °C) at a rate ≤3 °C/d. Juvenile plain pocketbook mussels were obtained from in‐house culture at the US Fish and Wildlife Service, Genoa National Fish Hatchery, approximately 1 mo before testing. Juveniles of the same cohort were used in each trial and thus varied in age and size at the time of testing. Animals in the 5 °C trial were 8 mo old (mean shell length 13.0, standard deviation [SD] 1.4 mm; *n* = 40), and those in the 20 °C trial were 16 mo old (mean shell length 17.2, SD 2.9 mm; *n* = 182). Plain pocketbook were placed into trays (11 cm diameter × 4 cm depth) that contained approximately 3.0 cm depth of washed sand substrate (Mastercraft® playground sand) and maintained in 30‐L tanks on the same water supply as the zebra mussels. Both zebra mussels and plain pocketbook were fed daily with a suspension of mixed algae (Nanno 3600, Shellfish diet 1800, W1200, TP1800; Reed Mariculture) to provide up to 6 mg L^−1^, based on dry weight. The algal stock was delivered continuously by a peristaltic pump to each holding tank at a rate of approximately 100 mL/h.

One week before the initiation of each temperature trial, zebra mussels and plain pocketbook were transferred from the holding tanks to a raceway to assess suitability for testing. Zebra mussels were sorted into “small” (8–14 mm shell length) and “large” (16–27 mm shell length) size categories. Groups of 25 to 30 small mussels and 25 large mussels were hand‐placed onto a conditioned acrylic plate (Plastikote® 12 cm length × 12 cm width, 0.32 cm thickness) that was then placed into a semirigid plastic mesh bag (3.0 mm mesh size) and transferred to the raceway. Plain pocketbook were removed indiscriminately from the holding tank and placed side‐lying into 24 trays (5 °C trial, *n* = 10; 20 °C trial, *n* = 9 per tray) that contained approximately 3.0 cm depth of washed sand substrate. Mussels were fed in the raceway with the same algal stock and ration as described in this section.

One day before CO_2_ exposure, the number of attached zebra mussels on each plate was counted to ensure a sample size of 15 to 25 mussels per size. Mussels were counted as “attached” if they had byssal thread attachments to the plate, mesh bag, or another mussel. Unattached zebra mussels were discarded. Four bags were transferred to each treatment tank. Trays of plain pocketbook were examined, unburied individuals were removed, and one tray was allocated to each test tank.

#### LC—CO_2_ exposure

Water quality (dissolved oxygen, pH, temperature) was measured at least once a day in each tank throughout the present study. Dissolved oxygen was measured with a YSI dissolved oxygen meter, and pH was measured with a ThermoScientific portable pH meter and probe (Orion Star A221). Temperature was measured with a digital thermometer. Conductivity and hardness were measured from one test tank per treatment at the beginning of each trial. Conductivity was measured with a Fisher Accumet conductivity meter (Fisher Scientific) calibrated against a standard solution (Rice et al. 2012). Total hardness (milligrams per liter of CaCO_3_) was determined by the titrimetric method with the Manver Red indicator (US Environmental Protection Agency 1983). Alkalinity was measured daily in the same tanks that were sampled for conductivity and hardness. Total alkalinity (milligrams per liter of CaCO_3_) was determined by the titrimetric method to a pH endpoint of 4.5 (Rice et al. 2012).

During CO_2_ exposure, one bag of zebra mussels was removed from each test tank at 24, 48, 72, and 96 h of exposure and immediately assessed. Attachment and narcotization (valves open and/or foot extended with reduced or no response to probing) were scored as 0 = negative or 1 = positive. The plate of zebra mussels was returned to the bag without disrupting byssal attachment and transferred to untreated water for 7 d postexposure. Food was delivered to test tanks during the exposure period and to the raceway during the postexposure period at the same ration as described in the section *LC—Test animals*. Attachment and narcotization were reassessed at 24 h postexposure; mortality and attachment were assessed at 7 d postexposure. Zebra mussel mortality was defined as lack of resistance when valves were gently pulled apart.

At the conclusion of each trial, a subsample of 80 zebra mussels per size was taken from control tanks to measure tissue condition. The soft tissue and shell were separated and placed into individual tared aluminum weigh pans. Shells were air‐dried, and tissues were oven‐dried at 60 °C to a constant weight (<1% change). Dry weights were measured on a Mettler model AT200 balance to the nearest 0.1 mg. Condition was defined as {[dry tissue wt (mg)/dry tissue wt (mg) + dry shell wt (mg)] × 100} (Davenport and Chen 1987).

Plain pocketbook were exposed to CO_2_ for 96 h. The number of mussels unburied (>90% of shell above substrate, side‐lying, or on umbo) in each tank was recorded daily during the exposure period. At 96 h, mussels were transferred to the raceway with untreated water for 7 d postexposure. Burial behavior was recorded during the postexposure period. Mortality was assessed at 7 d postexposure and defined as lack of resistance when valves were gently pulled apart or lack of response when probed.

### Time to lethality trials

#### LT—test system

Lethal time trials were conducted to determine the time required to produce complete mortality of zebra mussels when exposed to a set PCO_2_ at 3 temperatures. A PCO_2_ target range of 100 to 120 atm was chosen based on results of the LC trials in the present study and Waller and Bartsch (2018). The LT test system consisted of 2 headboxes (100 L) that each delivered well water to 4 test tanks (40 L). Tanks were filled to 28 L and received a continuous supply of temperature‐controlled well water at a rate of 500 to 640 mL min^−1^. Carbon dioxide delivery to the CO_2_ headbox was controlled by a Pinpoint CO_2_ regulator (American Marine). The control headbox was aerated with compressed air and delivered well water to 4 control tanks. The PCO_2_ in each tank was calculated as described in the section *LC—Test system*.

#### LT—Test animals

Zebra mussels were hand‐collected from White Bear Lake in October 2017. Plain pocketbook and fragile papershell were obtained from in‐house culture at the Genoa National Fish Hatchery. Plain pocketbook were approximately 17 mo old (mean shell length 15.2 mm, SD 2.1 mm; *n* = 246) and from the same cohort as those used in the LC trials. Fragile papershell were approximately 7 mo old (mean shell length 11.1, SD 1.6 mm; *n* = 264). Feeding and acclimation procedures are described in the section *LC—Test animals*. Mussels were held at the test temperature for a minimum of 10 d before testing.

One week before the initiation of each temperature trial, zebra mussels were transferred from the holding tanks to a raceway to assess suitability for testing, as described in the section *LC—Test animals*. Groups of approximately 25 zebra mussels (10–20 mm shell length) were hand‐placed onto plates, and plain pocketbook (*n* = 10) and fragile papershell (*n* = 11) were placed side‐lying into trays containing 3 cm washed sand. After assessment of test suitability, 4 bags of zebra mussels and one tray of each native species were allocated to each test tank.

#### LT—CO_2_ exposure

Time to 99% mortality (LT99) was estimated from reported results (Waller and Bartsch 2018) and the LC trials in the present study. The LT trials were conducted consecutively, from 2 January to 11 March 2018, in the order 12, 20, and 5 °C. The latter temperature was run twice, the second time with only zebra mussels, because of incomplete mortality in the first trial. Exposure duration was extended from 8 d in trial 1 to 12 d in trial 2. The results of both trials were included in the determination of LT99 calculations. Water quality (dissolved oxygen, pH, temperature) and water chemistry (hardness, alkalinity, conductivity) were measured by methods described in the section *LC—CO*_*2*_ *exposure*.

One bag of zebra mussels was removed from each tank starting 1 d before expected complete mortality and continuing until complete mortality was confirmed. After removal from the test tank, the bag was placed into the raceway with untreated water and held for 4 d postexposure in the 12 and 20 °C trials and 6 to 7 d postexposure in the 5 °C trial. Mortality was assessed on the last day of the postexposure period.

Unionid mussels were exposed to CO_2_ for the maximum duration of each trial, with the exception of trial 2 at 5 °C. Burial status was recorded once daily. At the end of the exposure, trays of unionid mussels were transferred to the raceway with untreated water for 4 to 8 d postexposure. Burial status and mortality were recorded on the final postexposure day.

### Statistical analysis

A generalized linear model (GLM) with binomial error distributions was used to model the effects of exposure duration, PCO_2_, and temperature on zebra mussel survival and attachment (Dalgaard 2008). The LC50, LC75, and LC99 values of zebra mussels were estimated from the model. Survival curves were plotted for plain pocketbook using gglplot2 (Wickham 2016), and exposure–response curves were estimated using the drc package (Ritz et al. 2015). The LC10, LC20, and LC50 values of plain pocketbook were calculated to determine the safety of CO_2_ treatments to the native species. A GLM with binomial error family and probit link function was also used to model the effects of exposure duration and temperature on survival and burial (unionids) at a constant PCO_2_ (LT trials). These results were used to adapt the model used by Nielson et al. (2012) with New Zealand mud snails for zebra mussels. A linear model was used to examine the effect of exposure duration, exposure treatment, and temperature on the mussels' condition (Dalgaard 2008).

Descriptive statistics (mean, standard error) were used to summarize water quality (temperature, dissolved oxygen, pH), water chemistry (alkalinity, hardness, conductivity), and PCO_2_. All analyses were done using R (R Development Core Team 2019) and summarized in an R Markdown document. Code has been provided at https://doi.org/10.5066/P9FMIHJM and includes the specific versions used as part of the saved output from the R Markdown file.

## RESULTS

### 24‐ to 96‐h LC trials at 5 and 20 °C

**Figure 1:**
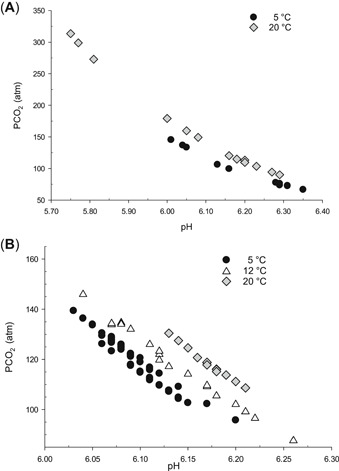
Partial pressure of CO_2_ and pH in (**A**) lethal concentration trials at 5 and 20 °C and (**B**) lethal time trials at 5, 12, and 20 °C. PCO_2_ = partial pressure of carbon dioxide.

Water chemistry (alkalinity, hardness, conductivity) was similar between the 5 and 20 °C trials (Supplemental Data, Table S1). Water temperature was similar among replicate treatment tanks (Supplemental Data, Table S2) in each temperature trial. Dissolved oxygen was inversely related to PCO_2_ in the 20 °C trial but remained above 6 mg L^−1^ in all test tanks during CO_2_ infusion. The dissolved oxygen concentration was consistent among treatments and test tanks in the 5 °C trial. In all trials, pH was inversely related to PCO_2_, reflecting the reaction of CO_2_ with water to form carbonic acid (Figure 1A).

The range of PCO_2_ in test tanks was lower in the 5 °C trial (45–160 atm) compared to the 20 °C trial (50–350 atm; Figure 1A; Supplemental Data, Table S2) and resulted in only partial overlap of PCO_2_ levels between temperatures. Therefore, we compared responses of mussels by measured PCO_2_ levels rather than categorical treatment levels (i.e., low to high). The variability in PCO_2_ within a treatment was relatively small at 5 °C, whereas within‐treatment variation was greater at 20 °C, particularly at higher PCO_2_ levels (Supplemental Data, Table S2).

Mean condition index of control zebra mussels differed between collection sites. Mean condition of zebra mussels from Lake Minnetonka (5 °C trial) was 5.6 (2.1 SD) and 3.9 (1.3 SD) in the small and large groups, respectively. In comparison, the mean condition of zebra mussels from White Bear Lake (20 °C trial) was 7.0 (1.4 SD) and 5.9 (1.1 SD) in the small and large groups, respectively.

#### Zebra mussel response

Zebra mussel mortality did not differ between size groups within each temperature (the regression coefficient for size was small 0.021, and the 95% CI included zero, –0.106 to 0.148). Therefore, size was removed as a factor in mortality models.

Mean mortality in control groups was 1.6% (SD 2.6, range 0–8.7%) at 5 °C and 0.6% (SD 1.6, range 0–5%) at 20 °C. In the 5 °C trial, mortality was minimal (4.5–28.6%) in all CO_2_ treatments after 24‐h exposure (Table 1; Supplemental Data, Figure S1). High levels of mortality occurred in the highest treatment (PCO_2_ = 160 atm) after 96‐h exposure (Table 1; Supplemental Data, Figure S1); however, because of incomplete mortality, the extrapolated LC99 values and wide confidence limits indicated large uncertainty in the values. Similarly, the 24‐h LC99 value for the 20 °C trial could not be determined with confidence. Mortality at 20 °C exceeded 75% after 24‐h exposure to PCO_2_ >300 atm and after 48‐h exposure to PCO_2_ >130 atm (Table 1; Supplemental Data, Figure S2). Mortality after 72‐ and 96‐h exposure was similar and ranged from 90 to 100% at PCO_2_ >104 atm. The LC50 values at 20 °C were 29% (48 h) to 48% (72 h) less than those at 5 °C. Temperature‐related differences in LC75 values were even greater and ranged from 37 (48 h) to 62% (72 and 96 h). The data indicate that 72‐ or 96‐h exposure to a lethal PCO_2_ (LC99) at 20 °C would kill just 50% of mussels at 5 °C.

**Table 1:** Lethal partial pressure of CO_2_ (95% CI) to produce 50, 75, and 99% mortality of zebra mussels at 2 temperatures and 4 exposure durations

Hours of exposure	LC50^a^ (atm)		LC75^a^ (atm)		LC99a,, b (atm)
	5 °C	20 °C	5 °C	20 °C	20 °C
24	NA	264 (245–282)	NA	349 (315–384)	>300
48	163 (147–179)	115 (112–119)	218 (186–251)	137 (132–143)	236 (211–260)
72	160 (141–180)	84 (82–86)	243 (194–292)	94 (91–96)	133 (124–141)
96	124 (111–137)	73 (71–75)	215 (171–258)	82 (79–85)	118 (109–127)

^a^ Estimates are based on mortality at 7 d postexposure.

^b^ Values of LC99 could not be estimated at 5 °C.

LC50, LC75, LC99 = 50, 75, and 99% lethal concentrations; NA = not available (value could not be estimated with confidence).

Detachment during CO_2_ exposure was slightly greater in small mussels compared with large mussels, but the difference was small, if present at all (the regression coefficient was 0.02, and 95% CI included zero, –0.11 to 0.15); therefore, size was removed as a factor in attachment models. Effective concentration to cause detachment of 50% of mussels (EC50) was similar at 5 and 20 °C (Table 2; Supplemental Data, Figure S3); however, the EC75 indicated that CO_2_ was more effective at 20 °C than at 5 °C for causing detachment after 48 h. During the postexposure period, mussels that were exposed for <96 h at 5 °C showed recovery from CO_2_ by reattaching in greater numbers (Supplemental Data, Figure S3). In contrast, mussels that were exposed for >24 h at 20 °C continued to detach during the postexposure period, a sign of latent mortality.

**Table 2:** Effective partial pressure of CO_2_ (95% CI) to cause 50 and 75% detachment of zebra mussels at 2 temperatures and 4 exposure durations^a^

Exposure duration (h)	EC50 (atm)		EC75 (atm)	
	5 °C	20 °C	5 °C	20 °C
24	173 (139–207)	217 (203–232)	NA	NA
48	105 (91–119)	125 (120–130)	NA	192 (180–204)
72	96 (85–108)	94 (90–97)	359 (262–455)	140 (133–147)
96	67 (60–74)	68 (64–73)	245 (196–294)	111 (106–117)

^a^ Estimates are based on attachment at the end of the exposure period.

EC50, EC75 = 50 and 75% effect concentrations; NA = not available (value could not be estimated with confidence).

**Figure 2:**
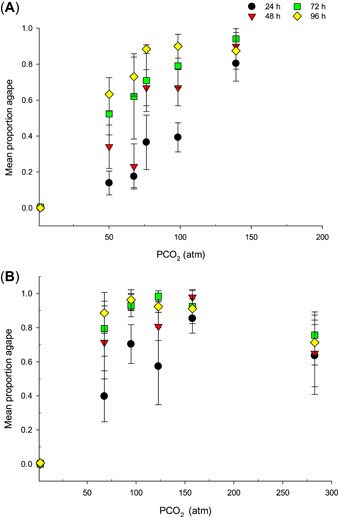
Gaping response of zebra mussels after exposure to CO_2_ at (**A**) 5 and (**B**) 20 °C. Mean partial pressure of CO_2_. Mean proportion agape (symbol) and standard deviation (error bars); *n* = 30 to 50 mussels in 4 replicate tanks. PCO_2_ = partial pressure of carbon dioxide.

Zebra mussels were narcotized (shells agape, no response to probing) at all levels of CO_2_. Gaping behavior at 5 and 20 °C was similar at comparable PCO_2_ levels. For example, the mean proportion agape in 140 atm was approximately 80 to 95% at 5 °C and 60 to 100% at 20 °C. At 5 °C, the proportion agape was correlated with PCO_2_ and, in part, with exposure duration (Figure 2A). At high PCO_2_ levels, 80% of mussels were agape at all exposure durations, whereas at lower PCO_2_, the proportion agape increased with exposure duration. At 20 °C, gaping response was similar across PCO_2_ treatments after 24 h (Figure 2B) and averaged >50%.

#### Plain pocketbook responses

In the 5 °C trial, plain pocketbook survival was 100% in all treatments. In the 20 °C trial, survival was near 100% at PCO_2_ <160 atm, except for one mortality each in medium (mean PCO_2_ = 118 atm) and medium‐high (mean PCO_2_ = 170 atm) treatments. High levels of mortality occurred in the 20 °C trial at PCO_2_ >274 atm. Only 16.7% of plain pocketbook in the high (mean PCO_2_ = 325 atm) treatment tanks survived compared to 97.2 to 100% in the lower treatments. The estimated 96‐h lethal concentrations (95% CI) of PCO_2_ for plain pocketbook in 20 °C water were LC10 = 173 (147–198), LC20 = 195 (172–219), and LC50 = 241 (217–265) atm PCO_2_.

**Figure 3:**
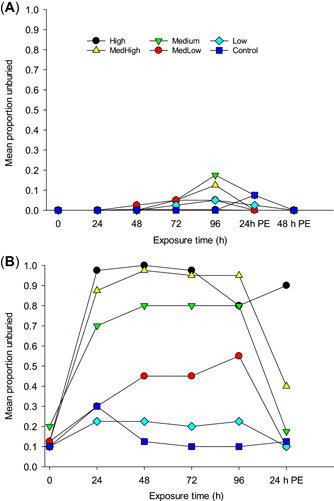
Burial response of plain pocketbook mussels after (**A**) 96‐h exposure to elevated CO_2_ at 5 and 20 °C and (**B**) after postexposure in untreated water; *n* = 9 or 10 mussels in 4 replicate tanks. See Supplemental Data, Table S2, for mean partial pressures in each treatment level. PE = postexposure.

Burial behavior of plain pocketbook also varied between 5 and 20 °C. On average, 20% were unburied at 5 °C after 96‐h exposure to medium and medium‐high treatments (Figure 3A), but >90% buried during the postexposure period. In 20 °C water, CO_2_ prompted mussels to move to the surface and unbury, except at the low concentration. At 24 h, >70% of mussels were unburied in medium to high treatments (Figure 3B). Mussels buried within the first 24 h of the postexposure period (Figure 3B). A video recording of mussels showed that most buried within 4 h after placement in untreated water (https://doi.org/10.5066/P9FMIHJM; Waller and Bartsch 2020).

### Lethal time to mortality

Water chemistry (alkalinity, hardness, conductivity) was similar among test tanks at the 3 test temperatures (Supplemental Data, Table S1). Dissolved oxygen was inversely related to PCO_2_ but remained >6 mg L^−1^ in all test tanks throughout the exposure (Supplemental Data, Table S3). Mean PCO_2_ ranged from 111 to 121 atm among all trials (Supplemental Data, Table S3). At a given pH, PCO_2_ was 20 atm less at 5 °C compared to 20 °C (Figure 1B).

#### Zebra mussel response

**Figure 4:**
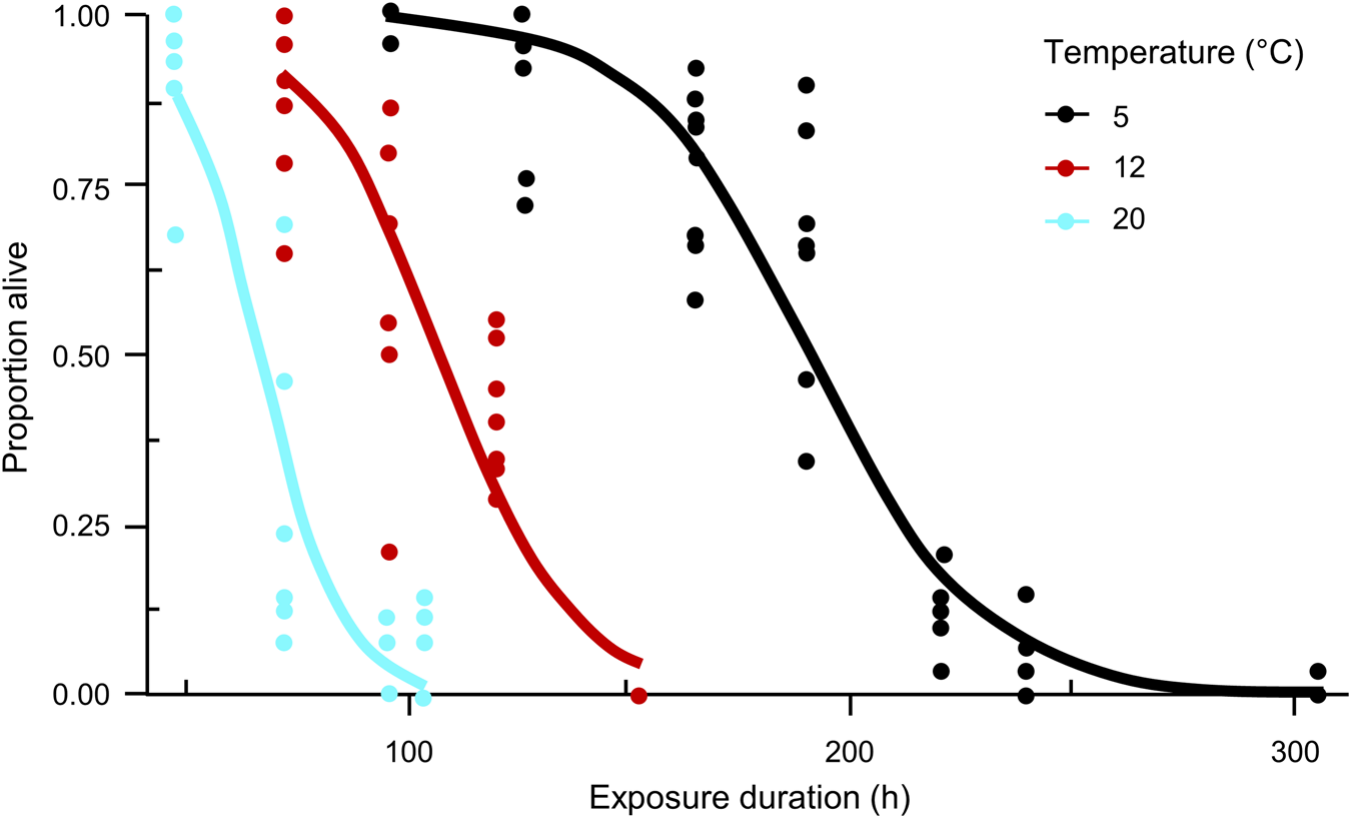
Survival probability of zebra mussels during exposure to CO_2_ (partial pressure of CO_2_ = 110–120 atm).

Mean mortality in control groups was 0% at 5 °C, 2.9% (SD 1.2%, range 0–5%) at 12 °C, and 0.1% (SD 0.8%, range 0–4.8%) at 20 °C. Lethal times to mortality were negatively correlated with water temperature (Figure 4; Supplemental Data, Table S4). The LT99 was approximately 100 h (4 d) at 20 °C compared to approximately 300 h (12 d) at 5 °C (Supplemental Data, Table S4). The results were then used to create a toxicity model similar to that of Nielson et al. (2012): Proportion alive = log10(Temperature × Exposure duration [h]). However, the data did not have a temperature and exposure product where mortality always occurred. Instead, 2 to 3 unique mortality patterns emerged when temperature was included. The survival curve at 5 °C differed from that for the higher temperatures (Figure 4). The survival curve declined most rapidly at 20 °C compared to an extended mortality period at 5 °C (Figure 4).

#### Two native mussel responses

Mortality of control mussels was <5% in all groups, except for plain pocketbook in the 5 °C group (Table 3). Mean mortality of this group was 20% (range 0–40%) and was greater than that of mussels in the CO_2_ treatment (mean = 12.5%, range 0–30%). Therefore, we did not consider data from plain pocketbook in the 5 °C trial when comparing CO_2_ effects on mortality. We found no detectable effect of CO_2_ treatment on mortality of plain pocketbook at 12 and 20 °C (Table 3). However, mortality of fragile papershell was greater in the CO_2_ treatment, and there was no detectable effect of temperature (Table 3).

**Table 3:** Mean percentage (standard deviation) unburied and mortality of plain pocketbook (*Lampsilis cardium*) and fragile papershell (*Leptodea fragilis*) exposed to partial pressure of CO_2_ (110–120 atm) in lethal time trials^a^

Treatment	Unburied (%)								
	5 °C		12 °C		20 °C		Mortality (%)		
	Exposure	PE	Exposure	PE	Exposure	PE	5 °C	12 °C	20 °C
Plain pocketbook (*n* = 10–11 per tank)						
Control	42.5	35.0	2.3	6.8	7.5	5.0	20.0	0.0	2.5
	(15.0)	(10.0)	(4.5)	(4.6)	(5.0)	(5.8)	(16.3)	(0.0)	(5.0)
CO_2_	52.5	45.0	32.3	7.0	10.0	5.0	12.5	9.5	5.0
	(35.0)	(17.3)	(20.9)	(4.7)	(14.1)	(5.8)	(12.6)	(8.2)	(5.8)
Fragile papershell (*n* = 11 per tank)						
Control	2.3	2.3	0.0	0.0	2.3	4.4	0.0	0.0	4.4
	(4.5)	(4.5)	(0.0)	(0.0)	(4.5)	(5.1)	(0.0)	(0.0)	(5.0)
CO_2_	37.9	24.2	52.3	13.6	20.1	9.3	11.1	13.6	7.0
	(25.7)	(15.7)	(15.5)	(11.7)	(8.9)	(7.4)	(17.2)	(5.2)	(4.7)

^a^ See text for duration of exposure and postexposure periods at each temperature.

PE = postexposure.

Burial response to CO_2_ treatment and temperature differed between plain pocketbook and fragile papershell (Table 3). In control tanks, fewer fragile papershell were unburied compared to plain pocketbook at all temperatures, whereas the reverse was true during CO_2_ treatment at 12 and 20 °C. More plain pocketbook were unburied in all tanks at both time periods in the 5 °C trial compared to the 12 and 20 °C trials. Burial status corresponded to greater mortality in the 5 °C plain pocketbook group and was likely indicative of a declining condition of these individuals. Both species showed evidence of recovery by reburying during the postexposure period (Table 3).

## DISCUSSION

### Zebra mussel responses

Dreissenid control efforts can occur across a range of water temperatures depending on management goals. Treatments may be applied in warm water (>15 °C) to optimize efficacy of the control product (Luoma et al. 2018; LimnoTech 2019) and target susceptible stages of the mussel (e.g., reproduction and early life stages; Costa et al. 2008; McCartney 2016). Cold‐water treatments may be warranted in rapid response to new infestations (Fieldseth and Sweet 2016; Lund et al. 2018) or within the thermocline of a large water body (Hammond and Ferris 2019). Cold water generally requires extended exposure and/or concentration of the product to be effective (Luoma et al. 2018). For example, lethal times to produce 100% mortality of zebra mussels with EarthTecQZ, a copper‐based biocide, extended from 10 d at 23 °C to 33 d at 5 °C (Hammond and Ferris 2019). The minimum lethal dose of potassium chloride to achieve 99% mortality of zebra mussels in 14 d decreased from >586 mg L^−1^ at 7 °C to 125 mg L^−1^ at 22 °C (Luoma et al. 2018).

We also found that zebra mussel mortality in CO_2_ treatments was temperature‐dependent and that efficacious exposure periods and/or PCO_2_ levels decreased with increased temperature (Table 1 and Figure 4; Supplemental Data, Figure S2). For example, mortality in 96‐h exposure to approximately 110 to 127 atm PCO_2_ was approximately 50% at 5 °C versus 99% at 20 °C. Similarly, the LC values were 1.4‐ to 2.6‐fold greater at 5 °C relative to 20 °C for a given exposure duration. In cold water, zebra mussel mortality did not expressly increase when exposure duration was increased from 48 to 96 h (e.g., LC75 values at 5 °C for 48–96 h exposure were similar). At 20 °C, LC values decreased with each additional 24 h of exposure. The Carbon Dioxide‐Carp label recommends 200 mg L^−1^ CO_2_ for a minimum of 96 h, but no maximum exposure is listed (US Environmental Protection Agency 2019). Our results indicate that under‐ice exposure for 12 to 14 d with 110 to 120 atm PCO_2_ could kill zebra mussels.

We proposed to develop a model of degree hours of exposure to achieve 100% mortality (Nielson et al. 2012) of zebra mussels at a constant CO_2_ level. However, the 5 °C mortality curve extended over a longer time period relative to that at 12 and 20 °C and did not fit the model. Data are needed at temperatures within the 5 to 20 °C range to determine whether degree‐hour models can be developed for discrete temperature intervals (e.g., 5–10 °C, 15–25 °C), rather than across a wide range.

Seasonal differences in the reproductive and metabolic conditions of zebra mussels could affect their sensitivity to CO_2_ (Kilgour and Baker 1994; Costa et al. 2008). We collected zebra mussels at the same time of the year but from 2 different sources for the LC trials at 5 and 20 °C and detected a difference in tissue condition; however, we could not determine whether condition influenced CO_2_ tolerance. Several studies have reported no correlation between zebra mussel tissue condition and toxicity endpoints (Kilgour and Baker 1994; Costa et al. 2008; Luoma et al. 2018) or heat tolerance (Elderkin and Klerks 2005). The source of mussel stock had a substantial effect on the toxicity of several biocides (Kilgour and Baker 1994; Luoma et al. 2018) and warrants consideration in efficacy trials with any toxicant, including CO_2_.

Biofouling by dreissenids adversely affects a variety of industrial and municipal water users, marinas and related structures, spawning shoals, and native mussel beds. Our results indicate that intermittent, short‐term (24–48 h) infusion of 100 to 200 atm PCO_2_ could reduce and prevent attachment of zebra mussels at a range of water temperatures. The EC50 estimates at 5 and 20 °C were similar and decreased with exposure duration (Table 2). Zebra mussel detachment (percentage) increased with PCO_2_ (100–300 atm) but was similar after 24‐ or 48‐h exposure at 12 °C (Waller and Bartsch 2018). Carbon dioxide also inhibits byssal thread production (McMahon et al. 1995; Payne et al. 1998; Waller and Bartsch 2018) and could prevent settlement of translocators and early life stages. Veliger settlement is reduced in water with pH <7.0 (Claudi et al. 2012a, 2012b), which we achieved with 20 atm PCO_2_ at 20 °C. Low‐level, intermittent infusion of CO_2_ could be an effective tool to prevent biofouling and new settlement of dreissenid mussels.

Pretreatment with a narcotizing agent, such as CO_2_, could increase the efficacy of a biocide by preventing the valve closure response in zebra mussels and increasing contact with the biocide (Elzinga and Butzlaff 1994). Zebra mussels gaped when exposed to CO_2_ within 24 h at all treatment levels and water temperatures. The proportion of gaping mussels was related to exposure duration rather than to CO_2_ concentration. At 20 °C gaping response was similar across PCO_2_ levels for exposures >24 h, whereas at 5 °C there was a slight increase in response with PCO_2_ level. Anecdotally, we observed gaping behavior within 6 h of CO_2_ infusion but did not determine a minimal exposure/concentration for onset of narcotization. Narcotization may be most effective for biocides that are lethal to gill and membrane surfaces, such as oxidizing agents and surfactants (Glomski 2015). However, narcotization could reduce the efficacy of biocides that are only toxic after ingestion if it also causes decreased filtration and feeding of zebra mussels.

### Native mussel responses

The rapid establishment of zebra mussels in unionid habitats led to the decline and extirpation of native species in areas throughout the Great Lakes (Schloesser and Nalepa 1994; Nalepa et al. 1996; Schloesser et al. 1998; Martel et al. 2001; Strayer and Malcolm 2018). The greatest impact to unionids reportedly occurs in the initial years of infestation when the invasive population explodes (Lucy et al. 2014). The population “boom” of dreissenids is often followed by a “bust” to more moderate (threshold) levels where zebra mussels and unionids may coexist. A selective control tool, such as CO_2_, could be used to manage dreissenids below threshold densities to reestablish native populations in extirpated habitats and reduce “boom”‐stage infestations.

We found CO_2_ treatment regimens at all 3 temperatures that were efficacious to zebra mussels and caused little to no mortality of juveniles of the 2 unionid species. Previous studies indicate that treatments that are nonlethal to juveniles are also likely to be safe to adult mussels of these species (Hannan et al. 2016b; Waller et al. 2017, 2018; Jeffrey et al. 2018a, 2018b). However, we used 7‐ to 17‐mo‐old juveniles, which may be less sensitive than newly transformed juveniles to some toxicants (Wang et al. 2007, 2010). Newly transformed juveniles were more sensitive to copper than 2‐mo‐old juveniles but not to ammonia in 96‐h exposures. Copper may elicit a valve closure response in older juveniles, allowing them to avoid exposure, whereas ammonia may not produce the same response (Wang et al. 2007). Because CO_2_ causes narcotization, mussels are unlikely to avoid exposure by valve closure, and toxicity may be similar across juvenile ages; however, further testing of additional life stages, including the larvae (glochidia) and brooding females, and of additional species is needed to expand the database on CO_2_ toxicity to native mussels.

The potential risk of a CO_2_ treatment to unionids at different times of the year will depend on both the toxicity of CO_2_ and the seasonal behaviors and metabolic activities of native mussels. In warm water, efficacious treatments for zebra mussels are achieved with less exposure time and CO_2_ concentration, which could also minimize native mussel mortality. For example, in 20 °C LT trials, complete mortality of zebra mussels occurred at 100 h with no CO_2_‐related mortality of either unionid species. The 20 °C 96‐h LC50 for zebra mussels (Table 1) was approximately 3.3‐fold lower than that for plain pocketbook (LC50 = 225 atm PCO_2_). However, there was only a 21‐atm difference between the upper and lower confidence limits of the 96‐h LC10 (173 atm, CI 148–198) for plain pocketbook and the 96‐h LC99 for zebra mussels (118 atm, CI 109–127). Unionid mortality could occur when CO_2_ is applied at temperatures >20 °C (Jeffrey et al. 2018b) or when dose and/or duration exceed target levels.

Warmer water temperatures also coincide with reproduction in most unionid species (McMahon 1991; Haag 2012). Unionids have a complex life cycle that includes a parasitic glochidial stage on specific host fish for transformation to a free‐living juvenile. A variety of specialized “lures,” glochidial packets (conglutinates), and behaviors have evolved in each unionid species to attract a host fish to the female, increasing the likelihood of successful glochidial attachment to the host (McMahon 1991; Haag 2012). Carbon dioxide is an effective deterrent for fish (Clingerman et al. 2007; Kates et al. 2012; Dennis et al. 2015) and could disrupt the interaction of mussel and host fish during the glochidial release period. Behavioral responses (i.e., gaping and unburial) of juveniles to CO_2_ were more frequent at 20 °C compared to 5 °C and could also increase their risk of predation or displacement. Hasler et al. (2017) found that plain pocketbook and fatmucket spent more time with open valves in elevated CO_2_, whereas giant floater (*Pyganodon grandis*) had the opposite response. Exposure to elevated CO_2_ also elicited physiological signs of stress, such as elevated glucose, decreased [Mg^2+^] and expression of heat shock protein 70, and increased oxygen consumption (Hannan et al. 2016a, 2016b; Jeffrey et al. 2017). Responses varied among unionid species and with the duration and concentration of CO_2_ exposure; however, unionids recovered quickly from CO_2_ exposure in warm water (17.5–22 °C), as evidenced by a return to baseline valve activity (Hasler et al. 2017) and hemolymph constituent concentrations (Hannan et al. 2016a, 2016b).

As water temperature decreases, efficacious CO_2_ treatments for zebra mussels require extended exposure, approximately 3 times longer at 5 versus 20 °C, and/or higher CO_2_ levels. The mortality of plain pocketbook in the LC and LT trials at 5 °C was mixed and appeared to be related to laboratory holding and handling in cold water, rather than CO_2_ treatment. In the LT 5 °C trial, plain pocketbook mortality in the control group was inadmissibly high (mean 20%, SD 16.3%) and exceeded that of the CO_2_ treatment group (Table 3), indicating that mortality was not related to treatment. Moreover, survival of plain pocketbook in the LC 5 °C trial was 100% in all treatments. There was no effect of CO_2_ treatment on mortality of fragile papershell in the LT 5 °C trial or of either unionid species in the LT 12 °C trial (Table 3). The LT 5 °C trial was the final one of the present study, and test animal condition may have declined during the laboratory holding period in this group. In the plain pocketbook LT control group, the percentage of unburied mussels was higher at 5 °C relative to 12 and 20 °C (Table 3), which further indicates that mussel condition was compromised in the latter group.

Positioning and locomotor behavior in unionids are temperature‐dependent (Waller et al. 1999; Block et al. 2013), and handling stress in cold water may have also contributed to mortality, especially in mussels with reduced condition. Adult plain pocketbook took longer to right themselves and moved less after disturbance at 7 °C compared to 21 °C (Waller et al. 1999). Pink heelsplitter (*Potamilus alatus*) failure to bury was significantly greater at 10 °C (66.6%) compared to at 30 °C (23.5%; Block et al. 2013). Early burrowing behaviors of this species (e.g., valve opening and foot extension) were significantly slower at 10 versus 30 °C, but burrowing performance was not. Conceivably, a greater number of plain pocketbook would have buried if the cold‐water acclimation period had been extended to allow more recovery time from handling.

In the wild, unionid mussels tend to bury in response to decreasing water temperatures (i.e., late fall to early spring) and reduce movements and the frequency of valve opening (i.e., reduced filtration; Amyot and Downing 1997; Watters et al. 2001; Lurman et al. 2014a, 2014b). Burial and locomotor movements of unionids vary widely among species (Waller et al. 1999) and are often related to reproductive activities (Watters et al. 2001). Cold‐water application of CO_2_ for dreissenid control corresponds to time periods when most unionid species are reproductively inactive and likely buried. The unburial response of mussels to CO_2_ also appears to be lessened in colder water. In LT trials, the proportion of unburied mussels during CO_2_ exposure was minimal at 5 °C (range 0.0–0.2) compared to the response at 20 °C (0.2–1.0). In general, exposure of unionid mussels to CO_2_ could be minimized if treatments are applied in cold water, after mussels have buried and filtration activity is minimized and before spring reproductive activities. No studies have measured the physiological and molecular responses of unionids to elevated CO_2_ in cold water to determine whether stress and energy demands are also reduced with water temperature.

Differences between unionid and dreissenid tolerance to hypercapnia may be tied to ionic composition and their capacity to regulate pH and calcium concentration. Unionids respond to elevated CO_2_ by increasing hemolymph [HCO_3_^−^] from shell storage, along with [Ca^2+^], and reducing Cl^−^/HCO_3_^−^ exchange at the gills (Byrne and Dietz 1997). Zebra mussels lack these mechanisms to reduce acidosis and to regulate [Ca^2+^] (McMahon 1996). Therefore, water buffer parameters (alkalinity, calcium, hardness) and conductivity may disproportionately affect the toxicity of CO_2_ to zebra mussels compared to unionids. Adult zebra mussel mortality was significantly correlated with pH and calcium in a study of 16 Ontario lakes (Hincks and Mackie 1997). The resulting model predicted high mussel mortality (>80%) in a pH range of 6.5 to 8.0 when [Ca^2+^] was <25 mg L^−1^. Conductivity influenced the toxicity of potassium chloride (Moffitt et al. 2016) and phosphoric acid (Claudi et al. 2012b) to dreissenids and could likewise alter CO_2_ toxicity. Claudi et al. (2012b) reported approximately 40% mortality of adult quagga mussels after 10‐wk exposure to pH 6.9 in lake water with conductivity of 300 to 360 µS cm^−1^; in a follow‐up study, adult zebra mussel mortality was minimal after 8‐wk exposure to a similar pH in water with 2‐fold greater conductivity (613 µS cm^−1^). Other water quality parameters that could influence CO_2_ chemistry and mussel physiology, such as dissolved organic matter (D'Amario and Xenopoulos 2015), degassing, and photosynthetic activity (Guasch et al. 1998), need consideration when determining lethal CO_2_ treatments for dreissenids.

## CONCLUSIONS

Carbon dioxide could be an effective, inexpensive tool for management of dreissenid mussels in select situations. Intake lines from dreissenid‐infested waters could be infused with CO_2_ to kill and/or reduce attachment of mussels. Efforts to control dreissenids in open water have focused on small‐scale, high‐value habitats (LimnoTech 2019) or areas of early infestation (Fieldseth and Sweet 2016; Barbour et al. 2018; Lund et al. 2018; Enders et al. 2019). In the aforementioned cases, the control agent was applied within a barrier to maintain an effective concentration of the molluscicide. A similar approach could be used for application of CO_2_ in high‐priority areas, such as boat docks, mussel beds, native mussel propagation sites, and fish spawning reefs. Carbon dioxide could be infused at a low level to deter mobile native species from the area before a containment barrier is erected to isolate the treatment. Large‐scale CO_2_ infusion systems to deter invasive fish are in development (Zolper et al. 2018) and could be modified for application at select sites. Under‐the‐ice application of CO_2_ may be a feasible option for reducing dreissenid populations in a small water body and has USEPA registration for this use (US Environmental Protection Agency 2019).

Dreissenid control treatments with CO_2_ in areas with native mussel populations need to consider efficacy and cost of the treatment with the risk to native mussels. Treatment in warm water can kill zebra mussels in less time and likely reduce veliger numbers but may have greater risk to unionids, especially if treatment corresponds to reproductive activity when females are more stressed. Warm water may also release CO_2_ into the atmosphere more rapidly and require almost continuous infusion to maintain lethal concentration. Treatments in cold water will require much longer exposure time but will off‐gas more slowly and may require only intermittent infusions to maintain target levels. Cool‐ and cold‐water treatments may be safer to unionids, assuming that mussels are not disturbed during the treatment. Carbon dioxide can also reduce biofouling across a temperature range and would likely prevent settlement of early dreissenid life stages at levels below those that cause adult detachment.

## Supplemental Data

The Supplemental Data are available on the Wiley Online Library at https://doi.org/10.1002/etc.4743.

## Disclaimer

Any use of trade, firm, or product names is for descriptive purposes only and does not imply endorsement by the US government.

## Author Contributions

M.R. Bartsch and D.L. Waller: study design and conduct, critical revision of manuscript; E.G. Lord: data collection and entry, proofing, summary, critical revision of manuscript; R.A. Erickson: data analysis and interpretation, critical revision of manuscript; D.L. Waller: writing–manuscript, critical revision of manuscript.

## Supporting information

This article contains online‐only Supplemental Data.

Supporting information

## Data Availability

The data in support of this publication are available at https://doi.org/10.5066/P9FMIHJM.
