# Supplementary material for: Temperature‐Related Responses of an Invasive Mussel and 2 Unionid Mussels to Elevated Carbon Dioxide
Source: Environ Toxicol Chem. 2020 Jun 27;39(8):1546–57. doi: 10.1002/etc.4743 (PMC7496913; doi:10.1002/etc.4743)
Supplement: Supplementary file 1 — Supporting information [file ETC-39-1546-s001.docx]

Table 1. Mean (standard deviation) water chemistry (alkalinity, hardness, conductivity) by trial; *n* = sample size

| Trial |  | Alkalinity (mg L^-1^ as CaCO_3_) |  | Hardness (mg L^-1^ as CaCO_3_) |  | Conductivity (µS cm^-1^) |
| --- | --- | --- | --- | --- | --- | --- |
| LC 5 °C |  | 142.1 (3.6)  *n* = 15 |  | 202.0 (5.3)  *n* = 3 |  | 531.3 (2.1)  *n* = 3 |
| LC 20 °C |  | 145.3 (5.7)  *n* = 9 |  | 195.9 (3.0)  *n* = 9 |  | 392.3 (1.5)  *n* = 3 |
|  |  |  |  |  |  |  |
| LT 5 °C trial 1 |  | 140.2 (2.0)  *n* = 8 |  | 200.0 (3.8)  *n* = 6 |  | 436.5 (3.5)  *n* = 2 |
| LT 5 °C trial 2 |  | 145.3 (2.4)  *n* = 6 |  | 202.1 (5.6)  *n* = 6 |  | 412.0 (2.8)  *n* = 2 |
| LT 12 °C |  | 143.3 (1.7)  *n* = 6 |  | 199.1 (2.9)  *n* = 6 |  | 422.0 (1.4)  *n* = 2 |
| LT 20 °C |  | 141.6 (0.9)  *n* = 7 |  | 198.6 (1.9)  *n* = 4 |  | 418.5 (2.1)  *n* = 2 |

LC = Lethal concentration; LT = Lethal time.

Table 2. Mean (standard deviation) temperature, dissolved oxygen (DO), pH, and partial pressure of carbon dioxide (PCO_2_) by treatment and temperature trial. Temperature, dissolved oxygen, and pH were measured daily on each tank. PCO_2_ values were calculated using CO2Calc program (Robbins et al. 2010)

| Relative treatment |  | Temperature (°C) | |  | DO (mg L^-1^) | |  | pH | |  | PCO_2_ (atm) | | |  |
| --- | --- | --- | --- | --- | --- | --- | --- | --- | --- | --- | --- | --- | --- | --- |
|  | | 5 | 20 |  | 5 | 20 |  | 5 | 20 |  | 5 |  | 20 |  |
| Control |  | 4.8  (0.5) | 19.9  (0.1) |  | 10.0  (0.5) | 7.3  (0.8) |  | 7.91  (0.02) | 7.93  (0.06) |  | 2  (0.1) |  | 2  (0.3) |  |
| Low | | 4.7  (0.4) | 20.0  (0.1) |  | 10.2  (0.4) | 7.2  (0.7) |  | 6.47  (0.02) | 6.41  (0.05) |  | 52  (2) |  | 70  (7) |  |
| MedLow | | 4.6  (0.3) | 20.0  (0.1) |  | 10.0  (0.4) | 7.1  (0.6) |  | 6.34  (0.03) | 6.24  (0.01) |  | 69  (3) |  | 103  (4) |  |
| Medium | | 4.7  (0.3) | 20.0  (0.1) |  | 10.0  (0.4) | 7.0  (0.7) |  | 6.29  (0.02) | 6.18  (0.02) |  | 77  (4) |  | 119  (6) |  |
| MedHigh | | 4.5  (0.2) | 20.0  (0.1) |  | 10.0  (0.4) | 6.8  (0.7) |  | 6.17  (0.01) | 6.02  (0.06) |  | 100  (4) |  | 171  (23) |  |
| High | | 4.7  (0.3) | 20.1  (0.1) |  | 9.9  (0.4) | 6.1  (1.0) |  | 6.02  (0.03) | 5.74  (0.03) |  | 139  (8) |  | 325  (22) |  |

Table 3. Mean (standard deviation) temperature, dissolved oxygen (DO), pH, and partial pressure of carbon dioxide (PCO_2_) in each LT (lethal time) temperature trial. Temperature, dissolved oxygen, and pH were measured daily on each tank (*n* = sample times). PCO_2_ was calculated using CO2Calc program (Robbins et al. 2010)

| Temperature trial |  | Temperature (°C) | | | |  | DO (mg L^-1^) | |  | pH | |  | PCO_2_ (atm) | |
| --- | --- | --- | --- | --- | --- | --- | --- | --- | --- | --- | --- | --- | --- | --- |
|  |  | | Control | | Treatment |  | Control | Treatment |  | Control | Treatment |  | Control | Treatment |
| 5 °C trial 1  (*n* = 8) |  | 5.1  (0.5) | | 5.3  (0.2) | |  | 12.5  (0.4) | 11.3  (0.2) |  | 8.17  (0.03) | 6.11  (0.02) |  | 1.0  (0) | 113  (6) |
| 5 °C trial 2  (*n* = 12) |  | 5.1  (0.4) | | 5.3  (0.5) | |  | 12.6  (0.2) | 10.8  (0.4) |  | 8.14  (0.03) | 6.10  (0.08) |  | 1.1  (0) | 121  (16) |
| 12 °C  (*n* = 8) |  | 12.4  (0.7) | | 12.3  (0.5) | |  | 9.5  (0.7) | 8.4  (0.5) |  | 8.04  (0.06) | 6.15  (0.08) |  | 1.0  (0.2) | 111  (19) |
| 20 °C  (*n* = 4) |  | 20.1  (0.2) | | 20.3  (0.2) | |  | 7.8  (0.1) | 7.1  (0.1) |  | 7.95  (0.03) | 6.17  (0.02) |  | 1.9  (0.1) | 119  (6) |

Table 4. Lethal times (95% confidence limits) to produce 50% (LT50) and 99% (LT99) mortality of zebra mussels (*Dreissena polymorpha*) in exposure to elevated partial pressure of carbon dioxide (110 to 121 atm PCO_2_)

| Water temperature (°C) |  | LT50 (h) |  | LT99 (h) |
| --- | --- | --- | --- | --- |
|  |  |  |  |  |
| 5 |  | 183 (179–188) |  | 305 (282–328) |
| 12 |  | 95 (89–100) |  | 202 (165–239) |
| 20 |  | 60 (57–63) |  | 102 (92–113) |

Figure 1. Diluter system used in lethal concentration (LC) trials. (A) Food grade CO_2_ was infused into (B) the first chamber of the diluter and reduced in concentration by ~20% in (C) each subsequent chamber. Five levels of CO_2_ and a control were tested in four replicate (D) test tanks. (E) headbox overflow to control tanks.

Figure 2. Proportion of zebra mussels (*Dreissena polymorpha*) that survived exposure to carbon dioxide (partial pressure of CO_2_ in atmospheres) after 24-, 48-, 72-, and 96-h exposure at 5 and 20 °C. Mortality was assessed after 7-d post-exposure in untreated water.

Figure 3. Proportion of zebra mussels (*Dreissena polymorpha*) that remained attached after exposure to carbon dioxide (partial pressure of CO_2_ in atmospheres) after 24-, 48-, 72-, and 96-h exposure at 5 and 20 °C. Attachment was assessed immediately after removal from the CO_2_ treatment.


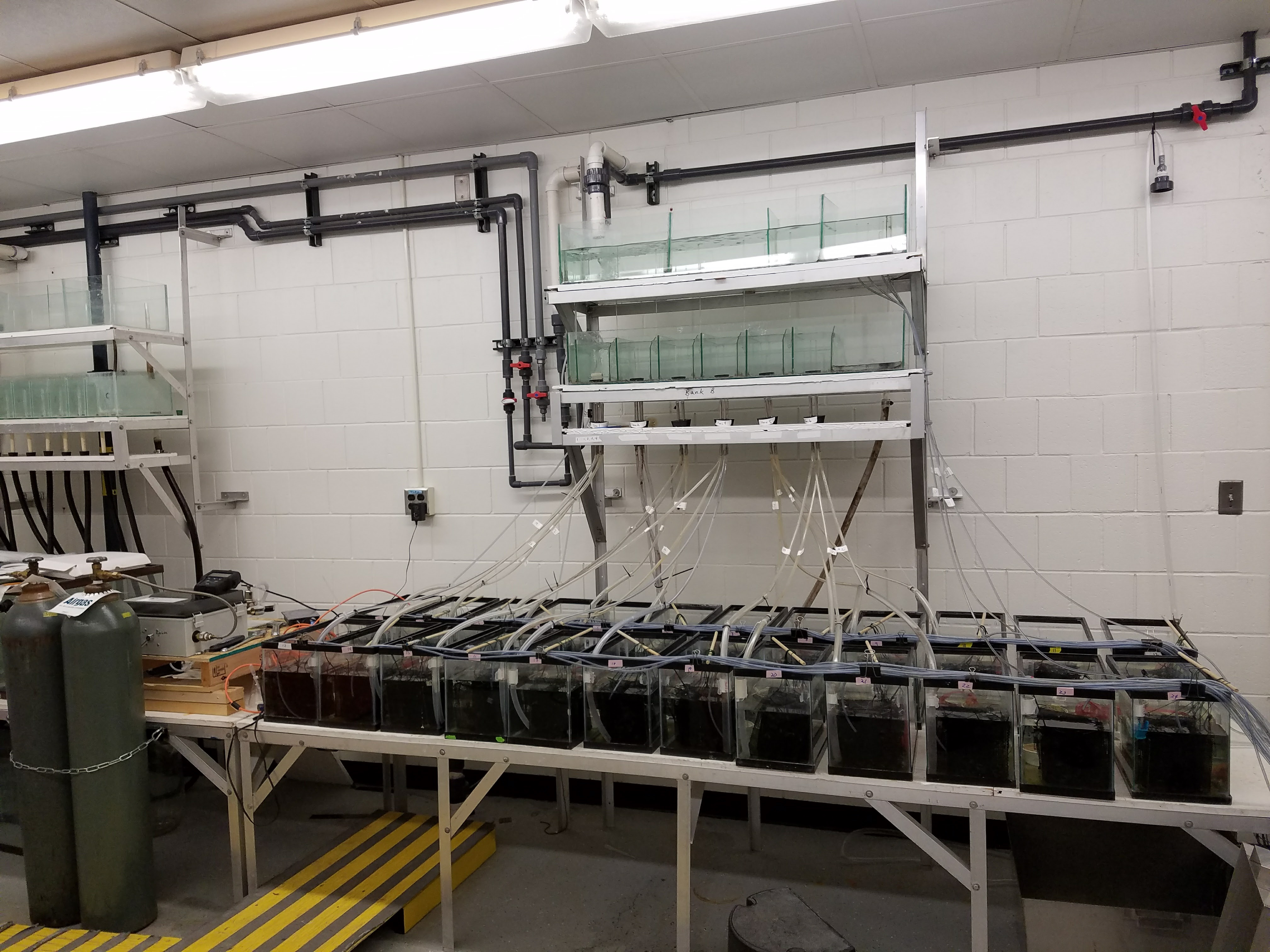


**C**

**E**

**D**

**A**

**B**


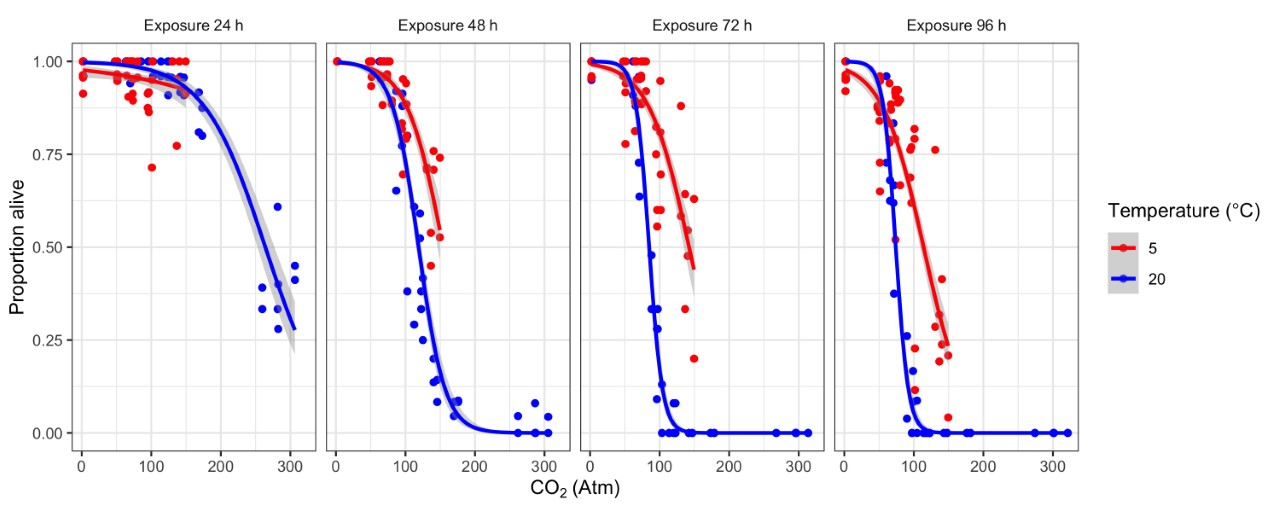


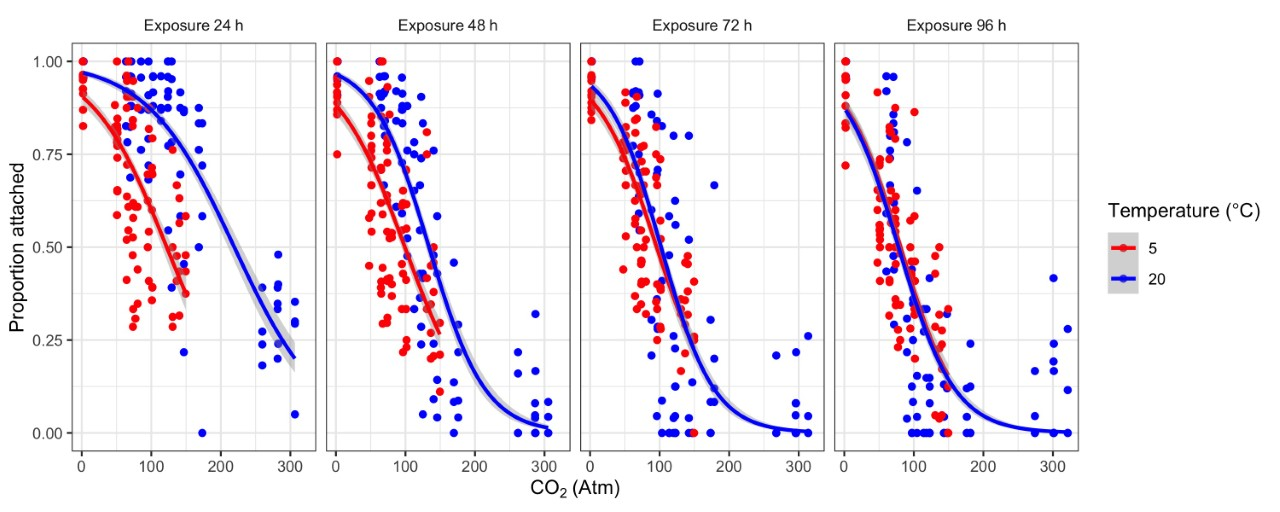


REFERENCES

Robbins LL, Hansen ME, Kleypas JA, Meylan SC. 2010. *CO2calc: A user-friendly seawater carbon calculator for Windows, Mac OS X, and iOS (iPhone)*. US Geological Survey. Open-File Report 2010-1280, 17 p.
